# The Ageing of μPlasma-Modified Polymers: The Role of Hydrophilicity

**DOI:** 10.3390/ma17061402

**Published:** 2024-03-19

**Authors:** Chang Che, Behnam Dashtbozorg, Shaojun Qi, Matt J. North, Xiaoying Li, Hanshan Dong, Michael J. Jenkins

**Affiliations:** School of Metallurgy & Materials, University of Birmingham, Birmingham B15 2SE, UK; b.dashtbozorg@bham.ac.uk (B.D.); sjqi886@gmail.com (S.Q.); mjn435@bham.ac.uk (M.J.N.); x.li.1@bham.ac.uk (X.L.); h.dong.20@bham.ac.uk (H.D.); m.j.jenkins@bham.ac.uk (M.J.J.)

**Keywords:** μPlasma, thermoplastics, hydrophilicity, wettability, ageing, modelling

## Abstract

Thermoplastic polymers exhibit relatively limited surface energies and this results in poor adhesion when bonded to other materials. Plasma surface modification offers the potential to overcome this challenge through the functionalisation of the polymer surfaces. In this study, three polymers of differing hydrophobicity (HDPE, PA12, and PA6) were subjected to a novel, atmospheric, μPlasma surface treatment technique, and its effectiveness at increasing the surface energies was evaluated via measurement of the contact angle. To characterise the physical and chemical changes following μPlasma surface modification, the surface morphology was observed using atomic force microscopy (AFM), and the functionalisation of the surface was evaluated using infrared spectroscopy. Immediately after treatment, the contact angle decreased by 47.3° (HDPE), 42.6° (PA12), and 50.1° (PA6), but the effect was not permanent in that there was a pronounced relaxation or ageing phenomenon in operation. The ageing process over five hours was modelled using a modified stretched exponential function Kohlrausch–Williams–Watts (KWW) model, and it was found that the ageing rate was dependent on the hydrophilicity of polymers, with polyamides ageing more rapidly than polyethylene.

## 1. Introduction

Thermoplastic polymers are deployed in a multitude of applications ranging from single-use packaging to their use in the automotive and aerospace industries. Engineering polymers such as polyamides (PAs) have gained widespread use in the automotive sector due to their desirable physical properties, such as high toughness, good thermal stability, mechanical strength, and impact resistance [1,2]. Polyamide 6 (PA6) is particularly popular due to its ductility, strength, and hot-forming properties [3]. Compared with PA6, which has a molecular backbone based on six carbon atoms, PA12 has longer methyl (CH_2_) chains and a lower melting point [4]. Its unique combination of properties, such as high chemical resistance, high pressure and impact resistance, and relatively low moisture absorption, make it an attractive material for various applications [4]. However, it should be noted that polyamides are more prone to moisture absorption, which can cause degradation during processing [3,5]. Commodity polymers such as polyethylene (PE) are extensively used as a flexible packaging material due to adequate flexibility and strength coupled with low cost [6]. Unlike polyamides, it is well-established that PE is a hydrophobic material with non-polar characteristics, thus making it a challenging polymer to wet or print on [7]. In general, polymeric surfaces are often characterised by poor wettability [8], which limits their potential in certain applications, such as joining dissimilar materials using adhesives.

To overcome this challenge and improve the versatility of polymeric materials in applications that require adhesive bonding, various surface modification techniques are commonly employed. Numerous studies have shown that plasma treatments are some of the most promising surface modification methods [9,10]. These techniques are capable of altering the physical and chemical properties of a material surface, thereby significantly enhancing the surface wettability and adhesion properties without influencing the bulk properties of the material [11,12,13,14]. Among the plasma treatments currently available, μPlasma surface modification is particularly interesting because, unlike traditional plasma treatments that require low-pressure conditions (typically 50–400 Pa) [15], μPlasma modifications can be performed under atmospheric conditions, thus significantly reducing the processing costs [16]. Given these advantages over conventional plasma treatment methods, μPlasma technology may enable future integration with robotic systems, thereby enabling precise, localised, and multi-axially adjustable μPlasma modification of geometrically complex industrial components.

Although it is well established that plasma surface treatment can change the surface morphology of a polymer and enhance surface wettability via the formation of new functional groups on the surface, it is not a permanent modification. Instead, the polymer surface is subject to post-treatment ageing phenomena. These ageing phenomena can be subdivided into degradative and non-degradative processes. Degradative processes may involve chain scission, crosslinking, and oxidation [17,18], whereas non-degradative processes may involve hydrophobic recovery that involves chain reorientation and diffusion back towards the bulk of the material. Previous studies have shown that post-treatment ageing is affected by temperature [19], relative humidity [20], and polymer crystallinity [21].

In this study, μPlasma surface modification of HDPE, PA12, and PA6 is explored. These polymeric materials were selected in order to assess the effect of hydrophilicity on the post-treatment ageing process. This study extends previous work via a decoupling of the effects of crystallinity and hydrophilicity on the ageing process. It also develops the characterisation of the ageing process via the application of a stretched exponential function to analyse and characterise the ageing data.

## 2. Materials and Methods

### 2.1. Materials

The materials utilised in this study are listed in Table 1. All were supplied in sheet form and were dried in a desiccator for 7 days prior to processing.

### 2.2. Sample Preparation and Crystallinity Control

The crystallinities of the polymers were varied via a controlled cool from the melt. This was carried out in a heated press (George E. Moore & Sons Ltd., Birmingham, UK).

**Figure 1:**
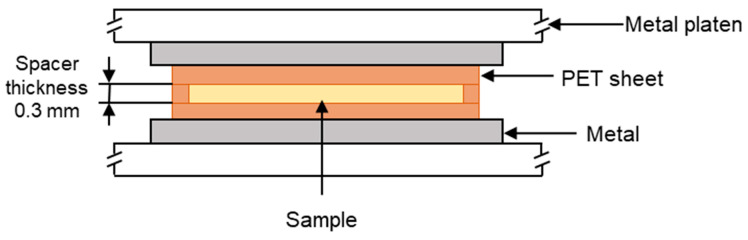
Cross section showing the hot-press arrangement (not to scale).

**Figure 2:**
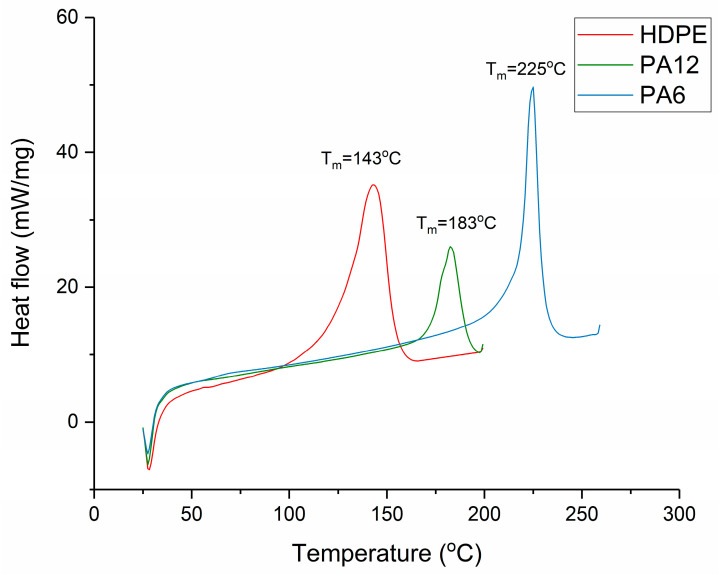
The thermal responses of HDPE, PA12, and PA6 recorded using DSC at a heating rate of 50 °C/min.

The polymer samples were placed into a polyethylene terephthalate (PET) mould that was composed of three sections. There was a PET spacer of thickness 0.3 mm, and on either side of this, there was a sheet of PET that functioned as a release film. This assembly was then placed in the preheated hot press, as shown in Figure 1. The polymers were heated for 3 min to initiate melting. Then, a load of 10 tonnes was applied via the platens for a further 5 min, causing the polymer melt to flow and then fill the mould. The polymers were then recrystallised by cooling from the melt under different cooling regimes, which are listed in Table 2. The melting temperatures of the ‘as received’ materials were determined using differential scanning calorimetry (DSC) as described in Section 2.3. The melting regions of each material are shown in Figure 2, and this informed the selection of the hot-press temperatures, which were as follows: 160 °C for HDPE, 200 °C for PA12, and 240 °C for PA6. The heat of fusion and the degree of crystallinity for each recrystallised sample were then determined via DSC (as per Section 2.3). Samples with comparable crystallinities were then selected for μPlasma treatment, and these are listed in Table 3.

### 2.3. Measurement of Crystallinity Using Differential Scanning Calorimetry (DSC)

A differential scanning calorimeter (PerkinElmer DSC-7, PerkinElmer Inc., Waltham, MA, USA) was used for determining the melting point and the degree of crystallinity of the cooled polymers. Disc-shaped samples were placed in aluminium pans and encapsulated with lids (aluminium 40 μL flat DSC pans and lids, Thermal Instruments Ltd., Lancashire, UK). The sample weight was 5 ± 0.1 mg. The samples were heated at 50 K min^−1^, and the melting point and heat of fusion were calculated. The resulting degrees of crystallinity were determined from the melting region according to a method described previously [22]. It is displayed in Figure 2 that the T_m_ values for HDPE, PA12, and PA6 were 143 °C, 183 °C, and 225 °C, respectively.

### 2.4. μPlasma Surface Modification

**Figure 3:**
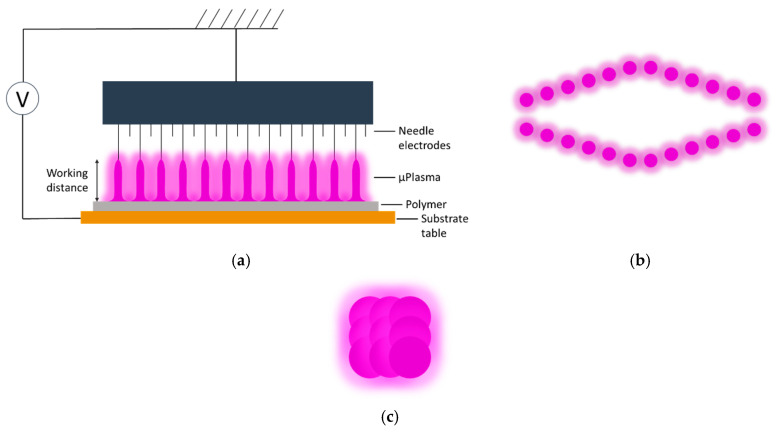
(**a**) Schematic of μPlasma modification setup, (**b**) top view of μPlasma discharge pattern generated by the 24 needle electrodes, and (**c**) μPlasma modification pattern. Reproduced with permission from [20].

Both the plaque and film polymer surfaces were cleaned using ethanol before μPlasma modification. The μPlasma modification was carried out using a Roth & Rau Pixdro LP50 plasma inkjet printer (InnoPhysics, Eindhoven, The Netherlands) with an InnoPhysics POD24 print-head as shown in Figure 3a,b. A printing rate of 20 mm/s, a working distance from the sample surface to the tips of the printing needles of 100 μm, and an accelerating voltage of 7 kV were chosen as the μPlasma modification parameters, which had previously been reported to induce the most significant enhancement in wettability [20]. Additionally, in this study, the polymer surfaces were modified using a single treatment scan. The plasma spots were designed to overlap with each other (shown in Figure 3c) to produce uniform treatment coverage.

### 2.5. Atomic Force Microscopy (AFM)

Surface morphologies were examined using an atomic force microscope (Bruker Multimode AFM, Billerica, MA, USA) operating with a silicon cantilever (Nanosensors PPP-NCHR, Neuchatel, Switzerland, tip radius < 10 nm, length 125 µm, resonant frequency 330 kHz) under a “tapping mode”. Before use, the cantilever was cleaned by rinsing it with ethanol and exposing it to a UV–ozone chamber for 10 min. Images were generated across an area of 16 µm × 16 µm, with a minimum of 3 different positions recorded for each type of surface. Three-dimensional height profiles, average roughness, and root-mean-square (RMS) roughness data were obtained from the AFM data using an open-source image processing tool (Gwyddion, SourceForge, San Diego, CA, USA). To limit the influence of macroscopic surface deviations (e.g., striations or bumps), three smaller areas (16 µm^2^) were chosen for each sample type to obtain comparable roughness measurements.

### 2.6. Measurement and Analysis of Contact Angles

The contact angles of the untreated and treated polymer surfaces were measured in accordance with the ISO 19403-2:2020 standard [23] using an experimental apparatus under the sessile drop method. The apparatus was adjusted to allow for samples to be placed in line with the camera and light source to ensure a clear and flat view of the droplet, which, therefore, enabled comparable measurements between polymers. An optimised measuring technique from our previous work was utilised [20]. Ionised water droplets with a volume of 6 μL were formed on sample surfaces using a calibrated pipette. Averages and standard deviations of contact-angle values were obtained using three repeat droplets for each sample type. Both left and right contact angles were measured for each droplet using the Ossila contact-angle measurement software (Version 4.1.4, Ossila Ltd., Sheffield, UK).

### 2.7. Phenomenological Modelling of the Post-Treatment Ageing Process

A stretched exponential function was used as the basis of a phenomenological model of the post-treatment ageing (or relaxation) process. The Kohlrausch–Williams–Watts (KWW) function has been applied to various relaxation phenomena in polymers, including dielectric relaxation [24], and in a slightly modified form, to enthalpy relaxation (or physical ageing) [25]. The basic stretched exponential function is shown in Equation (1), where the relaxation of the quantity $\emptyset \left(t\right)$ is given by

(1) $$\emptyset \left(t\right)=\exp\left[-{\left(\frac{t}{\tau }\right)}^{\beta }\right]$$

where *t* is the time, τ is the characteristic relaxation time constant, and β is the stretching exponent with a value between 0 and 1, which determines the shape of the ageing curve. To characterise the post-treatment ageing process, a modified KWW function was employed in which the change in contact angle (∆θ) varied with time according to Equation (1).

(2) $$∆\theta (t)={∆\theta }_{\infty }\left\{1-exp\;\left[-{\left(\frac{t}{\tau }\right)}^{\beta }\right]\right\}$$

The three unknown parameters, τ, $\theta_{\infty }$ (the contact angle when t approaches ∞), and β, were determined using non-linear least-squares fitting. The modification shown in Equation (2) is after that of Cowie and Ferguson [25].

### 2.8. Fourier-Transform Infrared Spectroscopy (FTIR)

The infrared (IR) spectra were obtained using a Nicolet MAGNA 860 FTIR spectrometer (Thermo-Scientific, Waltham, MA, USA) fitted with an attenuated total reflectance (ATR) accessory (Golden Gate ATR, Specac, Orpington, UK). The samples were μPlasma treated 10 times and then transferred instantly to the ATR (treated surface in contact with the ATR crystal). The spectral range was between 500 and 4000 cm^–1^. In total, 100 scans were recorded at a resolution of 2 cm^–1^.

## 3. Results and Discussion

### 3.1. Characterisation of the Hydrophilicity of PA6, PA12, and HDPE

To measure moisture uptake in the polymer samples listed in Table 3, they were dried in a desiccator for a period of one week, and then their weights were measured. Samples were then immersed in distilled water for 24 h, and their weights were measured once more. It can be seen in Table 4 that HDPE exhibited no water absorption (0% gain), revealing the hydrophobic nature of the material, whereas the weights of PA6 and PA12 increased 6.67% and 3.07%, respectively, indicating higher moisture absorption capability and a higher degree of hydrophilicity in the polyamides.

To support the above observation, the surface contact angles of three untreated polymer surfaces were measured, and the values are listed in Table 5. It is notable that HDPE exhibited a contact angle higher than 90° (92.9° ± 2.6°), indicating its hydrophobic surface, whereas the polyamides were found to have hydrophilic surfaces, as their contact angles were both lower than 90°. PA6 exhibited the lowest angle (75.9° ± 2.2°), suggesting the highest degree of hydrophilicity. Between HDPE and PA6, the surface contact angle of PA12 was measured to be 82.3° ± 1.8°, which also indicated a hydrophilic surface.

**Figure 4:**
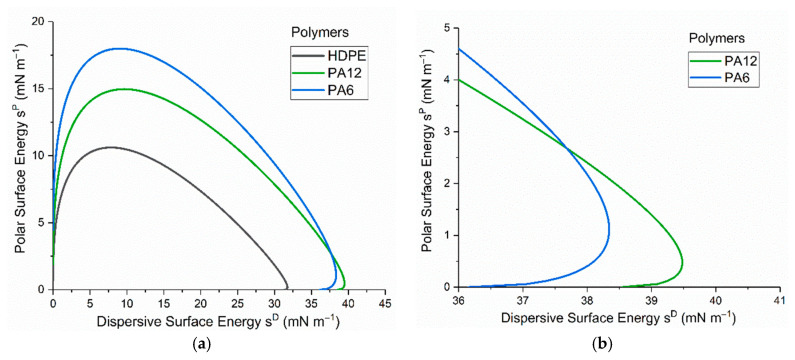
(**a**) Wetting envelopes representing liquids that should completely wet (i.e., 0° contact angle) untreated PA6, PA12, and HDPE surfaces, and (**b**) magnified views of PA6 and PA12 envelope overlap.

Using the Fowkes Equation [26], the surface free energies of the three untreated polymers were determined from the measurement of the contact angles formed with two liquids: deionised water and diiodomethane (shown in Table 5). As shown in Table 6, HDPE exhibited a total surface free energy of 32.1 mN/m, which was lower than both PA6 (40.6 mN/m) and PA12 (40.4 mN/m). Although the total surface free energies of PA6 and PA12 were found to be similar, the ratio of the polar components to the total surface free energy of PA6 was significantly higher (11%) than that of both PA12 and HDPE (4.7%) and (2.2%). The wetting envelopes shown in Figure 4 were determined using these surface free-energy values (the associated method is described in detail elsewhere [20]. Complete wetting (where the contact angle = 0°) can be expected when the values of polar and dispersive components of the liquid surface energy lie within the enclosed area of the wetting envelope. As shown in Figure 4, PA6 is clearly the most hydrophilic material in this study, but it is interesting to note that a liquid with higher polar surface energy is more likely to completely wet PA6, while a liquid with higher dispersive surface energy is more likely to completely wet PA12.

The moisture absorption capability and the calculated wettability data that are represented by total surface free energy and wetting envelope for the three polymers were in accordance with the water absorption capability. This is because the polyamide materials are inherently polar owing to the presence of polar amide groups (-C(=O)-NH-), which enables hydrogen bonding with water. As a result, polyamides exhibit a greater hydrophilicity. Polyethylene, on the other hand, is inherently nonpolar due to the presence of C-H and CH_2_ groups in the backbone.

### 3.2. Surface Morphologies of μPlasma-Modified PA6, PA12, and HDPE

**Figure 5:**
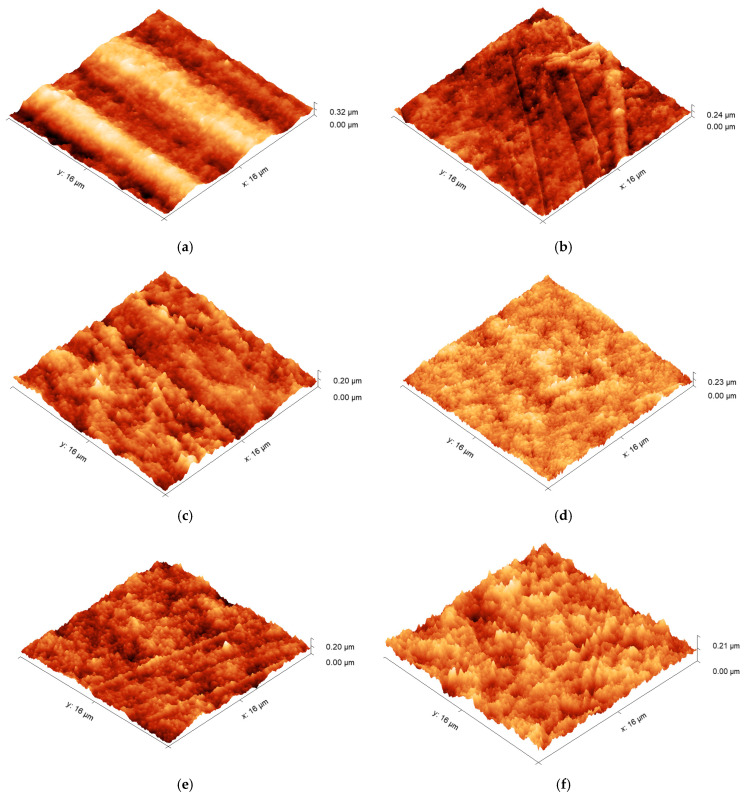
AFM 3D-view surface morphology images of (**a**,**b**) untreated and μPlasma-treated HDPE, (**c**,**d**) untreated and μPlasma-treated PA12, and (**e**,**f**) untreated and μPlasma-treated PA6.

**Figure 6:**
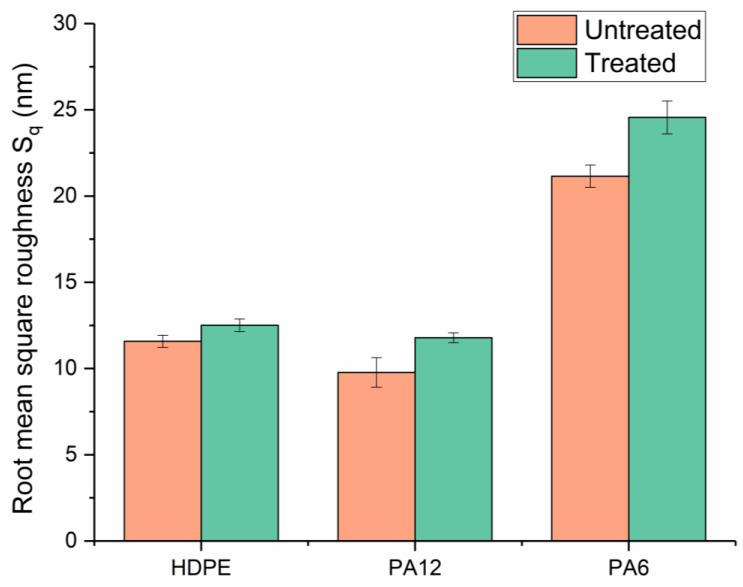
Root-mean-square roughness (S_q_) of untreated and μPlasma-treated HDPE, PA12, and PA6 surfaces sample surfaces.

The surface morphologies of untreated and μPlasma-treated materials (HDPE, PA12, and PA6) were investigated through the use of AFM, and the topographical images of the three polymer surfaces are displayed in Figure 5. It was apparent that following μPlasma treatment, the surfaces became more textured. To quantify the effect of μPlasma modification on polymer surface roughness, the root-mean-square (RMS) roughness (S_q_) of the three polymers was calculated; the results are illustrated in Figure 6. It was observed that the roughness of the HDPE surface increased following one treatment scan, increasing from an S_q_ of 11.6 nm (untreated) to 12.5 nm. Similar findings were observed for the polyamides, with S_q_ increasing from 9.8 nm to 11.78 nm for PA12 and from 21.2 nm to 24.6 nm for PA6, following μPlasma modification.

The increase in roughness after μPlasma modification is possibly due to the higher ablation rate of the amorphous phase compared to the crystalline phase on the semicrystalline polymer surfaces [27]. However, the changes were relatively insignificant, with RMS increases of 7.8% for HDPE, 20.2% for PA12, and 16.0% for PA6. This is attributed to the short duration of the single μPlasma modification, which was insufficient to produce a substantial difference in the roughness of the polymer surface.

### 3.3. Chemical Analysis of the Polymer Surfaces Using FTIR

**Figure 7:**
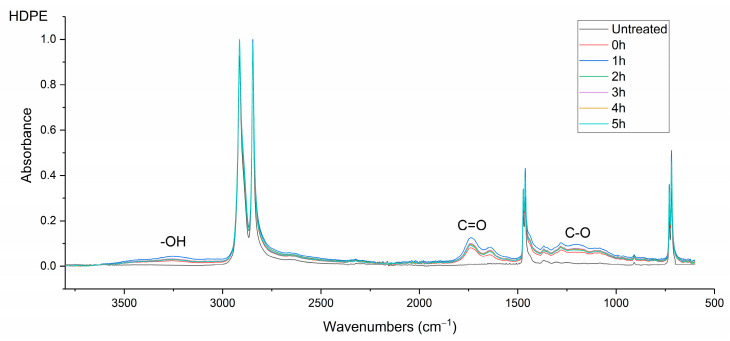
FTIR spectra of the untreated and μPlasma-treated (0–5 h aged) HDPE thin films.

The ATR-FTIR analysis revealed notable changes to the chemical composition of the polymer surfaces following μPlasma modification. Figure 7 shows the spectra of untreated HDPE and μPlasma-treated HDPE sampled every hour for 5 h. A broad band in the region of 3200–3500 cm^–1^ corresponding to hydroxyl groups (-OH) appeared following μPlasma modification. Two relatively narrow bands in the region of 1600–1750 cm^–1^, corresponding to the carbonyl group (C=O), and the region of 1085−1225 cm^–1^, related to the ether group (C-O), were also observed. Moreover, all the new peaks that appeared on the HDPE surface after the μPlasma modification were attributed to oxygen-containing polar groups, indicating that the functional groups on the HDPE surface reacted with the oxygen in the plasma during the treatment process. These newly formed polar functional groups are known to give higher hydrophilicity to the polymer surface [28,29]. However, over a period of five hours post-treatment, there was a decrease in the intensity of all treatment-associated bands.

**Figure 8:**
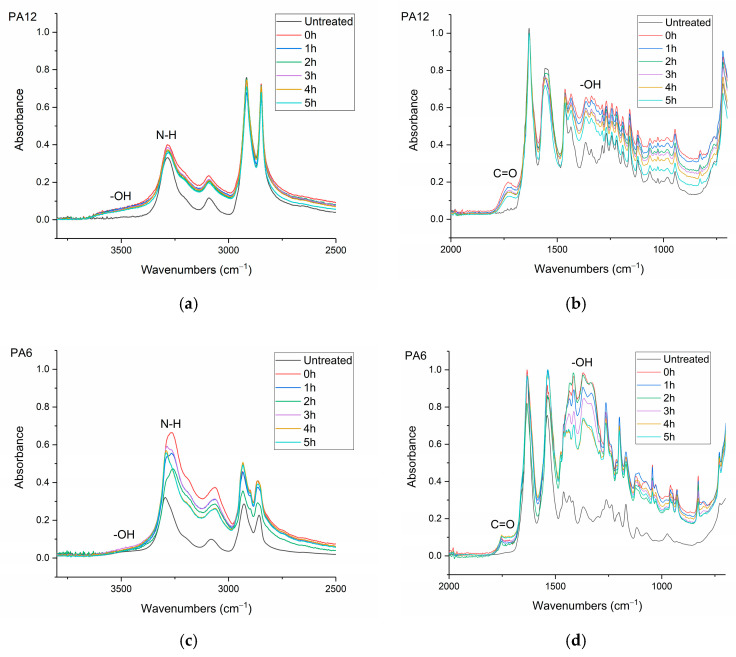
FTIR spectra of the untreated and 10 μPlasma-treatment scans over 0–5 h of ageing time: (**a**) PA12 3800–2500 cm^–1^, (**b**) PA12 2000–700 cm^–1^, (**c**) PA6 3800–2500 cm^–1^, (**d**) PA6 2000–700 cm^–1^.

FTIR spectra of untreated and μPlasma-treated polyamides aged for 5 h are presented in Figure 8a,b for PA12 and Figure 8c,d for PA6. Similar trends to HDPE were found following μPlasma modification, including the formation of a hydroxyl band (-OH) in the region of 3200–3500 cm^–1^ and a carbonyl band (C=O) in the region of 1600–1750 cm^–1^. In addition, there was increased intensity of the amide bands (N-H) in the region between 3000 and 3400 cm^–1^ and -OH bands in the region between 1330 and 1440 cm^–1^. This indicates a higher surface hydrophilicity of the polyamides following μPlasma modification, as all the increased bands are polar groups. The intensity of the peaks decreased over the 5 h of ageing (but over the timescale of this experiment, these new bands were retained, albeit at reduced intensities for all three materials). However, notably for PA6, the carbonyl (C=O) band increased with ageing time rather than decreasing like other bands (as shown in Figure 8d).

### 3.4. Variation in Contact Angle with Time Post-Treatment

Following a single μPlasma treatment scan, immediately after treatment, the measured contact angle was found to decrease for all three polymers (shown in Table 7). Reductions of 47.3° (50.1%), 42.6° (51.8%), and 50.1° (66.0%) for HDPE, PA12, and PA6 were found, respectively, confirming an immediate enhancement in wettability of the polymers.

It was noted in Section 3.3 that new polar functional groups were formed, and their intensity increased substantially immediately following μPlasma modifications for all three polymers. Conversely, the increases in roughness on the polymer surfaces were limited, with only 18.3% for HDPE, 25.9% for PA12, and 2.4% for PA6, as mentioned in Section 3.2. These findings indicate that the change in roughness is unlikely to be the dominant variable for the enhanced wettability of the polymers. In contrast, the effect of μPlasma on the chemical compositions was clear, especially for PA6. Therefore, it can be concluded that the change in chemical compositions attributed to the generation of the polar functional groups through μPlasma modifications is likely to play the dominant role in the change in the wettability of the polymer surfaces.

**Figure 9:**
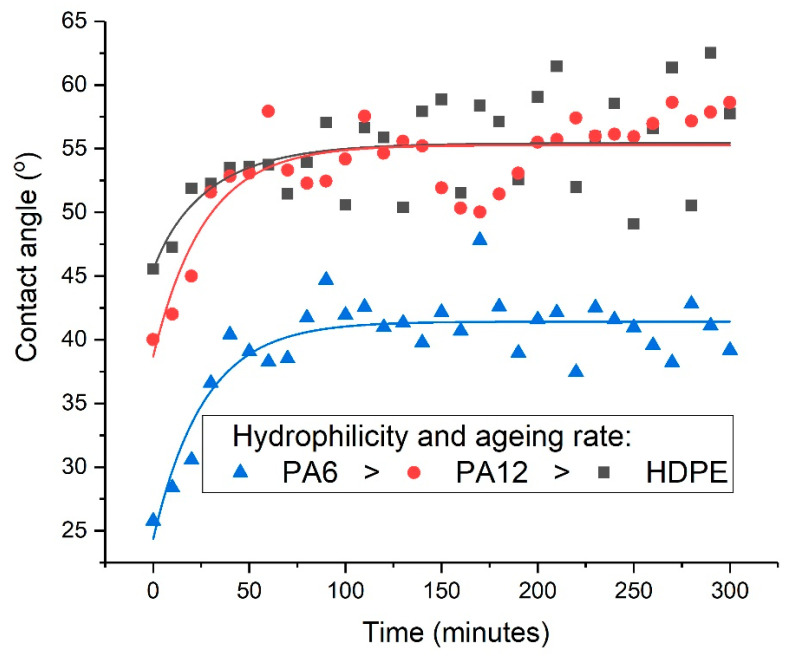
Contact angle variations of HDPE, PA12, and PA6 over 5 h of ageing following μPlasma treatment.

However, it was found that the initial improvement in the wettability was not permanent (at least under ambient atmospheric conditions), as the contact angles were found to increase significantly following μPlasma modification of all three materials, indicating a reduction in wettability. The variations in contact angle with time (ageing time) for each material are shown in Figure 9. The intensity of the polar groups, including hydroxyl (-OH), carbonyl (C=O), amide bands (N-H), and ether (C-O) groups, all decrease with ageing time following μPlasma modifications for the three polymers. This also suggests that the decrease in wettability corresponds to the decrease in polar functional groups. The behaviour of all three materials was similar in that after an initial rapid increase, the contact angle tended towards a plateau. However, it was noted that the relaxation/recovery of the contact angle in HDPE was weaker than in either PA12 or PA6. The contact angles eventually reach a plateau when hydrophobic recovery behaviour develops a balanced equilibrium.

To quantify these variations and yield a more accurate determination of the maximum predicted change in contact angle, the contact-angle data were fitted using Equation (2), and the parameters τ, $\theta_{\infty }$, and β were determined, as shown in Table 8. It is apparent that the stretched exponential function describes the ageing phenomenon adequately well, as evidenced by the R^2^ values.

As shown in Table 8, the shortest relaxation time τ was observed for PA6, followed by PA12. The longest relaxation time was found for HDPE, which took almost twice as long as PA6. The values of the predicted maximum contact angles $\theta_{\infty }$ are also shown in Table 8. Although the polymer surfaces had clearly aged on storage for five hours, the contact angles were not found to return to the original untreated values, indicating that the modified wettability was at least partially retained on all three polymer surfaces.

It was also found using Equation (2) that the maximum contact angle (the region where the curves reach a plateau and tend towards equilibrium values) increased by 27.6% for HDPE, 50.1% for PA12, and 68.2% for PA6 compared to their treated but unaged equivalents. This result strongly suggests that the relaxation time of the polymers is related to their hydrophilicities: the higher the hydrophilicity, the faster the relaxation time after the μPlasma modification. This can be attributed to the increase in chain mobility in the region of the surface caused by hydrogen bonding between water and the newly formed polar groups in this region of the material [5,30].

Generally, the newly formed polar groups tend to reorientate or diffuse back the bulk material to move away from a non-polar environment, such as air, which is known as hydrophobic recovery [31]. The higher hydrophilicity of a material facilitates the process of hydrophobic recovery. This is because the increased hydrophilicity of a material enhances the absorption of water molecules, which increases the free volume of the area in the region of the polymer surface, thereby enhancing the mobility of the chains and functional groups [32]. The increased chain mobility promotes the migration of these polar groups into the bulk of the sample and thereby results in an increase in the contact angle.

It was also noted in Section 3.3 that the carbonyl (C=O) band in PA6 increased rather than decreasing like other polar groups with ageing time (as shown in Figure 8d), therefore suggesting oxidation facilitated the ageing process of PA6 [33]. This could be another reason why PA6 experienced a faster ageing than the other polymers.

## 4. Conclusions

Surface contact-angle measurements, moisture absorption capability measurements, and calculations of wetting envelopes revealed that the hydrophilicity of the three polymers was in the order of PA6 > PA12 > HDPE, with HDPE being identified as hydrophobic. The wettability of the polymers was improved substantially through μPlasma modification, with increases in surface roughness and the introduction of more polar functional groups. The changes in chemical compositions of the polymer surfaces were believed to be the dominant factor for the improvement in wettability. Although the wettability enhancement of the three polymer surfaces following μPlasma modification was significant, it was subjected to an ageing phenomenon. The contact angle increased by 27.6% for HDPE, 50.1% for PA12, and 68.2% for PA6 following five-hour ageing, compared to their treated but unaged equivalents. However, the wettability was still partially remained. With the assistance of KWW modelling, it was found that the ageing rate was dependent on the degree of hydrophilicity of the polymers, i.e., the higher the hydrophilicity, the faster the ageing process. This finding was further confirmed by FTIR through the reduction in the polar functional groups, including hydroxyl (-OH), carbonyl (C=O), amide bands (N-H), and ether (C-O) groups.

## Figures and Tables

**Table 1:** Materials utilised in this study.

Materials	Producer
PA6 sheet	Goodfellow Cambridge Ltd., Huntingdon, UK
PA12 sheet	Aldrich, Merck Life Science UK Ltd., Gillingham, UK
HDPE sheet	Aldrich, Merck Life Science UK Ltd., UK

**Table 2:** Cooling regimes adopted for crystallinity control.

Cooling Rate	Description
CR1	Rapid extraction from the press and quenching into ice water.
CR2	Cooling in press with a flow of tap water circulating in the platens.
CR3	Removal from the press and cooled in air.
CR4	Cooling in press with open platens.
CR5	Cooling in press with closed platens.

**Table 3:** Degree of crystallinity in HDPE, PA6 and PA12.

Polymers	Crystallinity of the ‘as Received’ Material	Cooling Regime	Resulting Crystallinity Post-Cooling
HDPE	42.9% (±1.8)	CR1	34.3% (±1.7)
PA12	22.6% (±1.6)	CR5	32.3% (±2.0)
PA6	25.4% (±0.8)	CR4	33.8% (±0.8)

**Table 4:** Moisture absorption of PA6, PA12, and HDPE after immersion in water.

Polymers	Weight (Dry) ±0.01 g	Weight (Water Immersed for 24 h) ±0.01 g	Percentage Increase
HDPE	0.73 g	0.73 g	0.00%
PA12	0.02 g	0.02 g	3.07%
PA6	0.10 g	0.10 g	6.67%

**Table 5:** Contact-angle measurements of untreated PA6, PA12, and HDPE surfaces.

Polymers	Water Contact Angles	Diiodomethane Contact Angles
HDPE	92.9° ± 2.6°	55.1° ± 2.8°
PA12	82.3° ± 1.8°	41.1° ± 2.4°
PA6	75.9° ± 2.2°	30.7° ± 2.6°

**Table 6:** Surface free energies of untreated PA6, PA12, and HDPE.

Polymers	Total (mN/m)	Polar (mN/m)	Dispersive (mN/m)
HDPE	32.1	0.7 (2.2%)	31.4 (97.8%)
PA12	40.4	1.9 (4.7%)	38.5 (95.3%)
PA6	40.6	4.5 (11%)	36.1 (89%)

**Table 7:** Contact-angle measurements of μPlasma-modified PA6, PA12, and HDPE surfaces.

Polymers	Contact Angles (Untreated)	Contact Angles (Treated 0 h)
HDPE	92.9 ± 2.6°	45.6± 1.8°
PA12	82.3 ± 1.8°	39.7± 2.5°
PA6	75.9 ± 2.2°	25.8 ± 2.1°

**Table 8:** Kinetic parameters describing the relaxation of the contact angle during post-treatment ageing.

Polymers	Contact Angles (Untreated)	Contact Angles (Treated 0 h)	Relaxation Time τ	Stretching Exponent β	$\theta_{\infty }$	R^2^
HDPE	92.9°	45.6°	62.7 min	0.54	58.2°	0.59
PA12	82.3°	39.7°	54.9 min	0.33	59.6°	0.71
PA6	75.9°	25.8°	35.4 min	0.48	43.4°	0.77

## Data Availability

The data presented in this study are available on request from the corresponding author.
